# A Method for Comparing Proteins Measured in Serum and Plasma by Olink Proximity Extension Assay

**DOI:** 10.1016/j.mcpro.2025.101000

**Published:** 2025-05-27

**Authors:** Rawan Shraim, Caroline Diorio, Scott W. Canna, Erin Macdonald-Dunlop, Hamid Bassiri, Zachary Martinez, Anders Mälarstig, Afrouz Abbaspour, David T. Teachey, Robert B. Lindell, Edward M. Behrens

**Affiliations:** 1 Division of Oncology, Department of Pediatrics, Children's Hospital of Philadelphia, University of Pennsylvania Perelman School of Medicine, Philadelphia, Pennsylvania, USA; 2 Department of Biomedical and Health Informatics, Children's Hospital of Philadelphia, University of Pennsylvania Perelman School of Medicine, Philadelphia, Pennsylvania, USA; 3 Division of Rheumatology, Department of Pediatrics, Children's Hospital of Philadelphia, University of Pennsylvania Perelman School of Medicine, Philadelphia, Pennsylvania, USA; 4 Department of Medical Epidemiology and Biostatistics, Karolinska Institute, Stockholm, Sweden; 5 Division of Infectious Disease, Department of Pediatrics, Children's Hospital of Philadelphia, University of Pennsylvania Perelman School of Medicine, Philadelphia, Pennsylvania, USA; 6 Division of Critical Care Medicine, Department of Anesthesia and Critical Care, Children's Hospital of Philadelphia, University of Pennsylvania Perelman School of Medicine, Philadelphia, Pennsylvania, USA

**Keywords:** clinical proteomics, proximity extension assay, serum protein measurement, plasma protein measurement, data normalization, Olink

## Abstract

Accurate measurement of secreted proteins in serum and plasma is essential for understanding mechanisms and developing reliable biomarkers. Recent technological advancements, such as proximity extension assay (PEA), have enabled high-throughput multiplex protein analyses from small sample volumes in either serum or plasma. Despite the increasing use of PEA-based proteomics and the generation of extensive datasets, integrated data from these two mediums remains challenging due to inherent differences in sample processing. To address this issue, we developed and validated protein-specific transformation factors using linear modeling to normalize protein measurements between serum and plasma proteins quantified using Olink. Our analysis surveyed 1463 proteins across matched serum and plasma samples, identifying 686 transformation factors. The transformation factors were further validated using independent datasets generated from patients with different disease phenotypes and ages, and 551 of the models and transformation factors were reproducible. These transformation factors provide a valuable resource for normalizing PEA-based proteomic data across serum and plasma, ultimately enhancing the capacity for collaborative analyses and facilitating comprehensive insights across diverse disease phenotypes.

## Highlights

• Pearson correlation showed that most proteins have linear serum-plasma relationships.
• Developed linear models to transform Olink(R) serum-plasma data.
• Greater protein variant improved modeling of serum-plasma relationships in datasets.
• Validated transformation factors in diverse pediatric and adult cohort conditions.
• Identified 551 proteins with reproducible transformation factors across all cohorts.
• Scalable method enables cross-study proteomic data integration and biomarker discovery.

## Graphical Abstract

**Figure:**
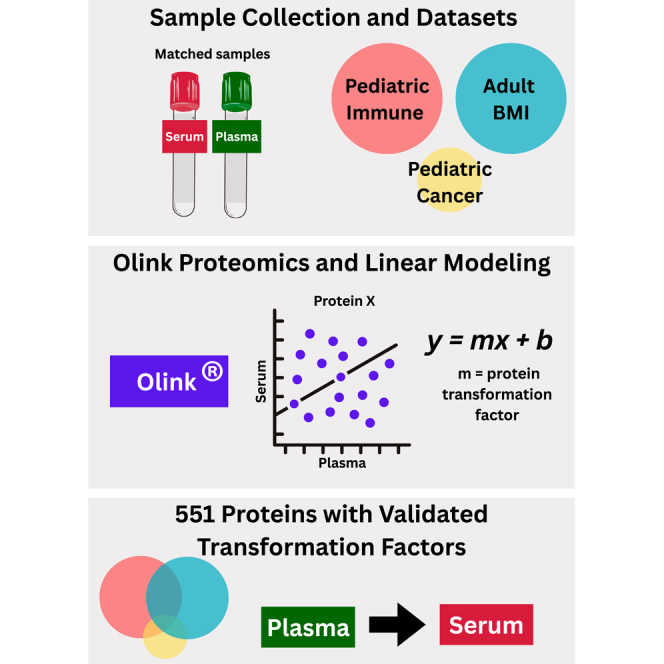

## In Brief

We developed and validated a methodology to normalize proteomic measurements between serum and plasma using the Olink proximity extension assay (PEA) technology. Using linear models, we generated transformation factors for 551 proteins that demonstrated robust, cohort-independent relationships across pediatric and adult datasets. This approach enables researchers to integrate serum and plasma datasets, expanding cohort sizes and supporting biomarker discovery, particularly in studies with limited sample availability.

Secreted proteins, integral to numerous physiological processes, are dynamically regulated in response to various stimuli such as inflammation, infection, and disease (1, 2, 3). Their release into the bloodstream initiates intricate cellular interactions, underscoring the importance of accurately measuring circulating proteins for elucidating disease mechanisms (3, 4, 5, 6, 7). In clinical and research settings, blood samples are commonly processed into serum or plasma to analyze disease biomarkers (5, 7, 8, 9). Serum, obtained from coagulated blood without additives, and plasma, obtained by anticoagulation followed by centrifugation, serve as valuable mediums for protein measurement (4, 9). The choice between serum and plasma varies based on practical considerations, technological specifications, and research field (9, 10, 11). While the ideal scenario is to collect both types of blood media, in pediatric research particularly, limitations on blood volume collection and cost of processing significantly impact sample collection strategies (5, 10, 11).

In recent years, technical advances have allowed for high-throughput multiplexing to enhance the search for blood-based proteins (5, 10, 12, 13, 14, 15). Within the proteomics field, affinity-based measuring platforms such as SomaLogic and Olink have become popular in measuring protein levels (12, 13, 15, 16, 17). The proximity extension assay (PEA) technique developed by Olink has been developed to translate protein information into expression values using small sample quantities from patient serum or plasma (12, 18). PEA involves the use of matched pairs of oligonucleotide-labeled antibodies that will bind to a specific target in a pairwise manner (19). Following the binding, the oligonucleotides are brought in proximity of each other, and a PCR target sequence is created, amplified, detected, and sequenced (19). During PCR amplification, given that the amplification is applied to the sample directly prior to sequencing, the number of reads will be relative to the total quantity of protein present thus decreasing bias in the sequencing data (19). To increase the high-throughput screening of the technology, PEA is combined with next-generation sequencing (NGS) to produce parallel sequencing for short reads.

The availability of the PEA-based proteomic technology has overcome the disadvantage of high sample quantity and quality required by proteomic methods such as mass-spectrometry, with the limitation that proteins are surveyed in a targeted panel (15, 20). Within the last 5 years, groups have used such assays to study protein profiles of many different diseases, such as obesity, cancer, and Alzheimer’s disease, making important insights that impact understanding of disease biology and clinical care (11, 20, 21, 22, 23). Our group has previously published on the protein profile of pediatric patients diagnosed with multisystem inflammatory syndrome (MISC) due to SARS-CoV-2 and on cytokine release syndrome (CRS) in pediatric B-cell acute lymphoblastic leukemia (B-ALL), identifying biomarkers associated with each disease-specific clinical phenotype (24, 25).

The widespread adoption of PEA-based proteomic technologies has led to an abundance of proteomics data available within collaborative teams; however, despite being processed using the same technology and the consistency in sample collection and processing protocols, a challenge persists in integrating these data due to differences in the measurement medium (6, 11, 26, 27). While both serum and plasma have been utilized for protein measurement, conventional practice has discouraged comparing or equating protein measurements due to variations in downstream processing. Comparative analyses of metabolites conducted by various groups in the metabolomics field have yielded differing results (27, 28, 29). Some studies have revealed substantial 18-fold differences between serum and plasma metabolites, while others have indicated minimal to no differences (6, 27, 28, 29). More recent studies show a high correlation between serum and plasma metabolite values; however, they do not attempt to model or transform the values between the two mediums (28). Additionally, while research has been conducted in assessing quantitative differences in values between serum and plasma proteins in metabolomics, no efforts have been made to assess differences in protein values in PEA-based technology or in the creation of methodologies to normalize serum to plasma-based data or vice versa.

The absence of a methodology to compare and transform serum to plasma protein values in PEA-based proteomic technologies poses a significant barrier for researchers aiming to leverage the wealth of available PEA-based proteomics data within their groups. It also imposes limitations on data that could be generated using banked samples that are collected and stored in different mediums. This limitation not only impedes effective collaborations within research teams but also restricts the integration of valuable patient data for cross-disease phenotype comparisons. To address this gap, our study collected matched serum and plasma samples from independent patient cohorts and measured proteins from both mediums using a high-throughput PEA-based proteomics method. We developed models to identify transformation factors to normalize serum and plasma proteomic quantifications within the applied technology, hypothesizing that comparability between serum and plasma would vary by protein, with some proteins being directly comparable between the two mediums. Our overarching goal is to enable the comparison of data from various studies and to develop a tool that can be utilized by us and other investigators to compare and integrate proteomic data generated using PEA technology.

## Experimental Procedures

### Study Design, Population, and Clinical Categorization

Written informed consent was obtained from all subjects or their legal guardian according to the Declaration of Helsinki, and all protocols were approved by their respective institutional review boards (IRBs) at the Children’s Hospital of Philadelphia. Patients from the original and the first validation cohort were enrolled in an institutional review board-approved biospecimen repository through the immune dysregulation program at the Children’s Hospital of Philadelphia.

For the MISC and COVID samples, the study was conducted in accordance with the Declaration of Helsinki and received approval from the Institutional Review Board (IRB) at the Children’s Hospital of Philadelphia (24). Verbal consent for this minimal risk study was obtained from patients or their legally authorized representative. Consent forms were signed by the consenting study team member and a copy was provided to the study participant or legally authorized representative. If appropriate, assent was obtained from children who were 7 years of age or older. Participants were not compensated for participation. For remnant samples obtained from healthy controls, protected health information (PHI) was not recorded. A limited chart review of this cohort was granted by the CHOP IRB to determine that patients met criteria to be considered healthy. CHOP IRB granted exemption criteria per 45 CFR 46.104(d) 4(ii) and waiver of HIPAA authorization.

### Sample Collection

Samples collected across the original and validation cohorts from the Children’s Hospital of Philadelphia were drawn and processed using similar protocols. Blood was drawn by phlebotomists or from central lines. Lithium heparin, mint top, and tubes were used to collect plasma samples, and red top tubes were used to collect serum samples. No additives were added after initial collection. Tubes were always processed within 24 h and typically <6 h after collection. Tubes were stored at −80 °C after processing. There was no variation in collection and processing across biospecimen sets. Samples from our original cohort were collected between 2020 to 2021, and samples from our validation cohort were collected from 2013 to 2020. Freeze-thaw cycles were minimized, and no samples experienced more than two freeze-thaw cycles. Details and procedures for sample collection for the second validation cohort can be found in PMID: 34715355 (19).

### Proteomic Measurements and Analyses

PEA proteomics, Olink, was used to measure proteins from serum and plasma samples to characterize the proteomic profiles of patients with various immune dysregulation states and diseases. The Olink Explore 1536/384 and the Olink Immuno-oncology 96 Target panels were specifically used to obtain proteomics data. For the original cohort, the Olink Explore 1536/384 was run at the Olink facility in Boston, and the Olink Target 96 Immune-Oncology panel used for the first validation cohort was run in Dr Diorio’s lab at the University of Pennsylvania. All analyses were completed using R and packages developed within R. For linear models, the ‘**lm**’ package was utilized. For Pearson correlations, the ‘**stats**’ package was utilized.

## Results

### Developing Linear Models to Identify Transformation Factors

**Fig. 1:**
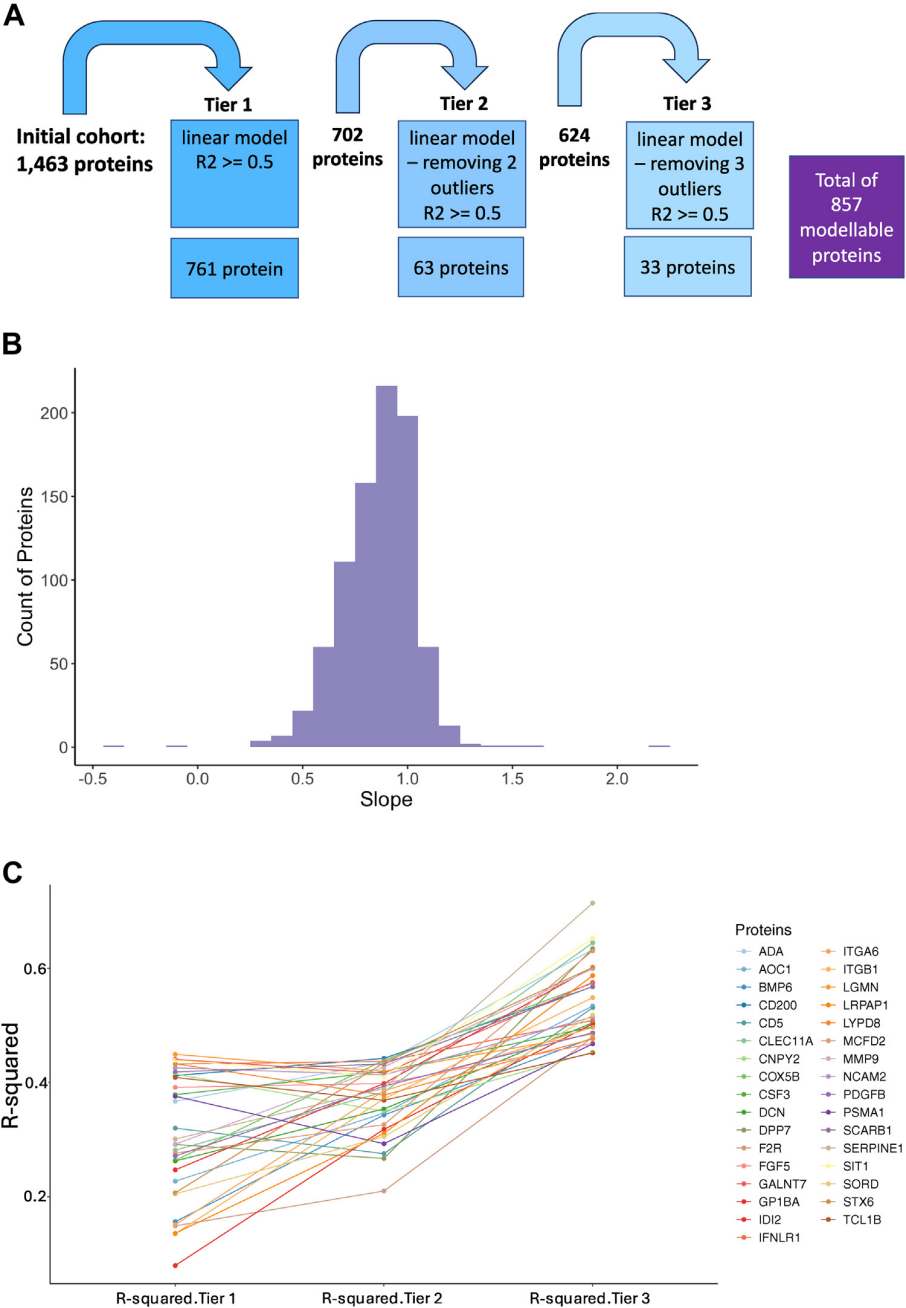
**Linear model development**. *A*, the linear model tier scheme developed to identify the linear relationship between serum and plasma protein Olink values. Outliers were defined by removing datapoints that have the highest Cook’s distance calculated based on the Tier 1 linear model. *B*, slope value of all the modelable proteins. *C*, the r-squared of improvement of the 33 proteins modeled in Tier 3.

The Olink Explore panel was used to measure 1463 proteins from 19 matched serum and plasma samples collected from a heterogeneous group of pediatric patients with monogenic, polygenic, and non-genetic immune-mediated diseases. This clinical diversity enabled a broad dynamic range of protein quantification levels in the data (supplemental Tables S1 and S2). To identify analytes with linear relationships between plasma and serum measurements, the Pearson correlation coefficient was calculated. Using a correlation threshold of ≥0.5, 885 proteins (60%) were identified as having moderate to strong linear behavior, supporting the hypothesis that linear modeling can effectively describe the relationship of these proteins. Recognizing potential outliers that would disrupt a linear relationship, we used the Cook’s distance of each data point in the linear model to identify influential points and remove these outliers in a systematic and unbiased way. To preserve as much of the data as possible while improving model fit, we implemented a tiered modeling approach using specific thresholds for data reduction, progressively excluding the most extreme outliers across three tiers, as shown in Figure 1*A*. Specifically, Tier 1 included all samples in the linear model, Tier two removed the top two outliers (representing a ∼10% data reduction) from the data and then fit a linear model to the remainder of the samples, and Tier 3 removed the top three outliers (representing a ∼15% data reduction) from the data then fit a linear model to the rest of the samples. A protein was defined as “modelable” if the R-squared value of the linear model was greater than or equal to 0.5. Thus, “modelable proteins” are ones in which there is sufficient correlation between plasma and serum values to allow for the consideration of computing a scaling factor between the two as the slope of the linear model. Proteins were fit into lower tiers of linear models only if they did not pass the 0.5 R-squared threshold of the tiers above it. This tiered strategy allowed us to balance model robustness with data retention and minimize the impact of outliers without significantly reducing the sample size.

### Characteristics of Modelable Proteins

Linear modeling was applied to all proteins in the cohort. Out of the quantified 1463 proteins, 761 proteins were modeled under Tier 1, an additional 63 proteins were modeled under Tier 2, and 33 additional proteins were modeled under Tier 3, leading to a total of 857 modelable proteins (supplemental Table S3). In all, 499 protein models (58%) had slopes that ranged from 0.75 to 1, as shown in Figure 1*B*. Assessing the R-squared of the linear models of proteins that were only modeled in tier 3, it was evident that removing the outliers produced linear models with higher R-squared values, as expected and shown in Figure 1*C*.

**Fig. 2:**
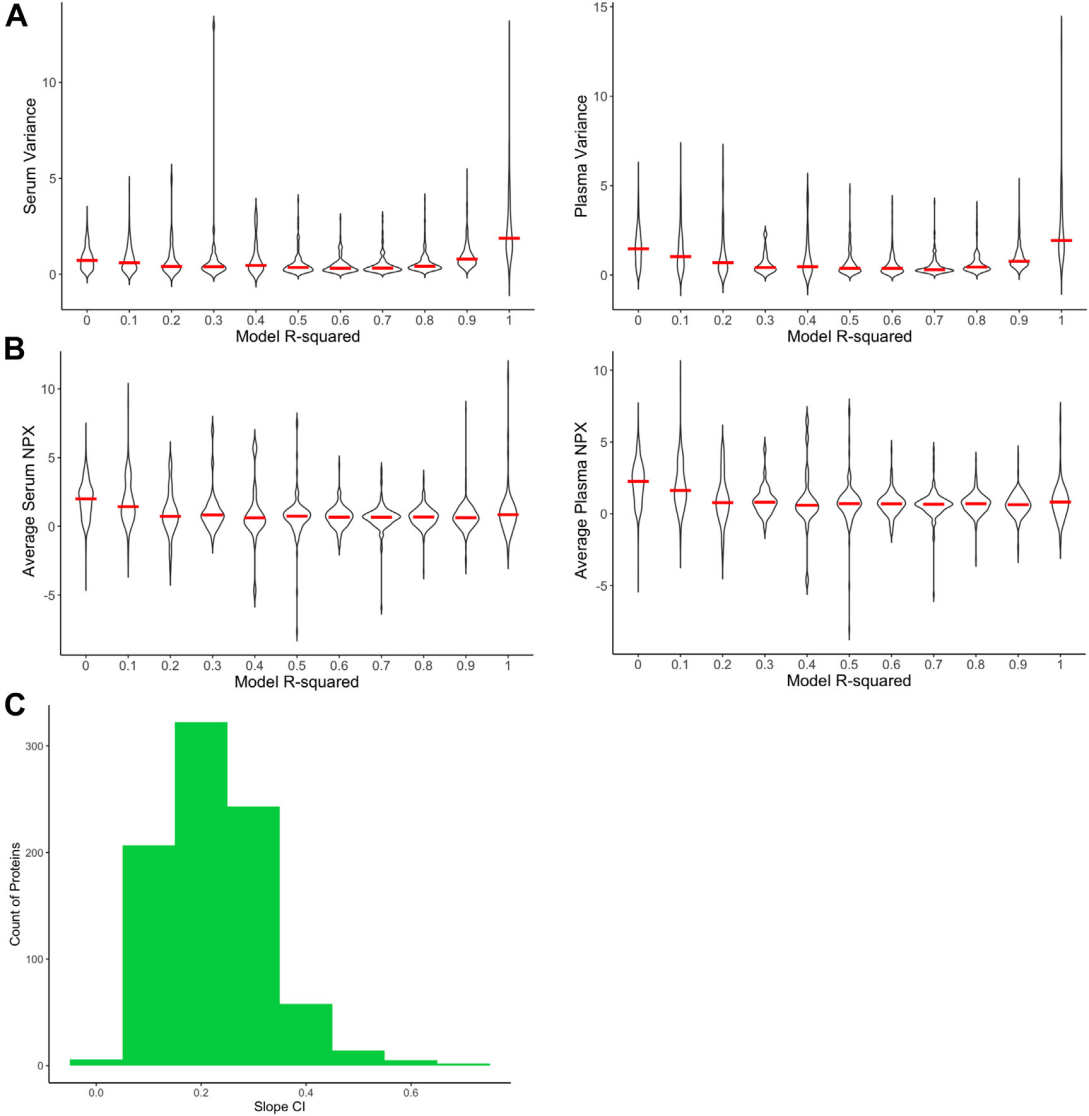
**Parameter characteristics of the initial cohort**. *A*, variance increased for proteins as their R-squared of the model was higher. *B*, NPX protein value did not determine modelling of protein and how well-fitted (r-squared) the model. *C*, the confidence interval (CI) of the slope for modelable proteins.

We hypothesized that some proteins might fail to be modelable due to insufficient variance within the cohort samples. To test this, we examined the relationship between protein variance and the corresponding R-squared values, as shown in Figure 2*A*, suggesting that proteins with an R-squared≥0.9 have higher variance, facilitating the generation of more robust models. We also explored whether protein expression levels influenced modeling capability, finding no significant relationship between expression levels and the ability to model proteins, as shown in Figure 2*B*.

### Transformation Factor Confidence Intervals and Sensitivity Analysis

The slope of the linear model provides a transformation factor that allows for scaling between serum and plasma protein values, while the intercept reflects cohort-specific scaling and can be disregarded for general conversion. The degree to which a slope can accurately define a transformation between serum and plasma will be reflected in its confidence interval (CI). To understand the potential accuracy of the values in our model, we calculated the CI of the final slope assigned to each protein using the standard deviation produced by the model. The histogram of the CI’s of the slopes, Figure 2*C*, shows that 95% of the modelable proteins CI’s range between 0 to 0.4.

To define an allowable CI range for the transformation factor for proteins, we performed a sensitivity analysis using a publicly available independent Olink dataset that looked at healthy controls and compared them to pediatric patients who were infected with SARS-CoV-2 and developed MISC or minimal COVID symptoms (24). Using the entire dataset, protein values were transformed by 0.1 intervals between 0.6 and 1.4, and a differential expression analysis (DEA) was completed between groups of patients within that study. Significantly differential proteins (Adj.p.value ≤ 0.05, Foldchange ≥2) that came out the analyses were compared at each interval change to the original list of differentially expressed protein. Proteins that held their significance and direction of change in both the modified and the original data lists were labeled as true-positive (TP), proteins that were only significant in the modified analysis or had a difference in their direction of change were labeled as false-positive (FP) and proteins that were significant in the original DEA list but lost significance in the modified analysis were labeled as false-negative (FN).

**Fig. 3:**
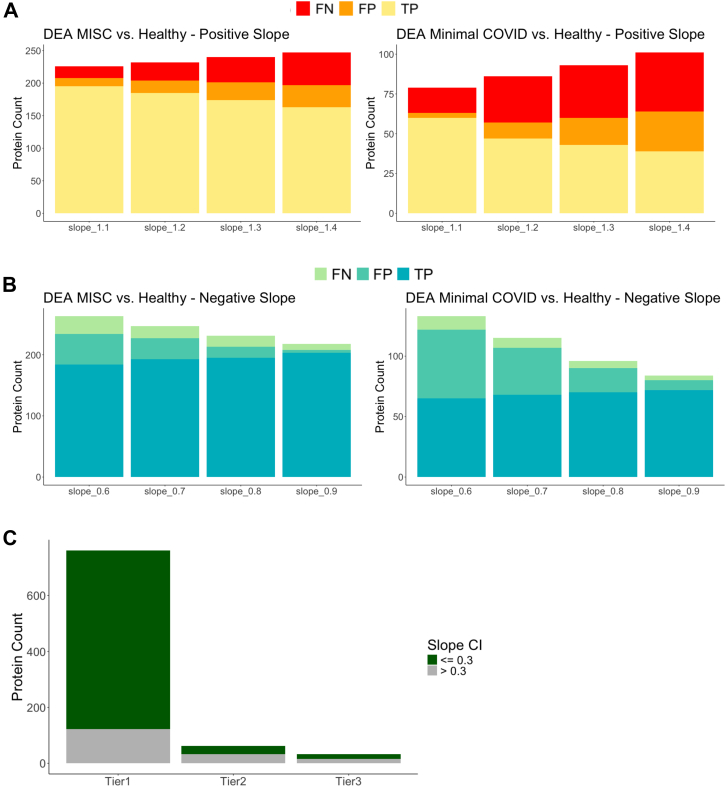
**Sensitivity analysis using COVID Olink proteomics dataset.***A*, varying transformation values in the positive direction between 1 to 1.4 at 0.1 intervals, negative direction between 0.6 and 1.4 at 0.1 intervals, examining MISC and healthy patients’ subset of patients. Proteins that held their significance and direction of change in both the modified and the original list were labeled as true-positive (TP), proteins that were only significant in the modified analysis or had a difference in their direction of change were labeled as false-positive (FP) and proteins that were significant in the original DEA list but lost significance in the modified analysis were labeled as false-negative (FN). *B*, varying transformation values in the positive direction and negative direction between 0.6 to 1.4 at 0.1 intervals, comparing minimal COVID and healthy patients. *C*, majority of the proteins had a slope CI between 0.1 to 0.3.

Initially, the healthy group of patients was compared to the MISC group of patients in the dataset, as shown in Figure 3*A*. From the original analysis, 212 proteins were significantly different between these two groups (24). Post-modifying of the protein values in the positive direction, at the highest interval of 1.4, 17% of the differential proteins were labeled as FP, and 23% of the initial differential proteins were missing and labeled as FN. At the highest change interval, 77% of the initial findings were retained. As shown in Figure 3*A*, as the interval increased, so did the number of FN in the dataset. In the negative direction, the rates of FP increased as the interval decreased, as shown in Figure 3*A*. At the interval of 0.6, 17% of the differentially expressed proteins were labeled as FP and 13% were labeled as FN, retaining 87% of the initial findings. Overall, this result showed groups that had vastly different biology were reasonably resistant to variances in transformation factors up to 0.4 units from the true value.

As a secondary analysis, we compared the patients within the cohort that only experienced minimal COVID symptoms to the healthy patients. From the original analysis, a total of 75 proteins were identified as significantly different between both subsets (24). Looking at the differentially expressed proteins post-modifying in the positive direction, 40% of the significant proteins were FP, 48% were labeled as FN, and thus retaining only 52% of the initial findings at the 1.4 slope interval. In the negative direction at the 0.6 interval, 47% of the differentially expressed proteins were labeled as FP, 13% was labeled as FN, and 87% of the initial findings were retained. Comparing the two analyses, the minimal COVID versus healthy patients’ comparison accumulated double the percentage of false-positive labeled findings at the highest change intervals. This result shows that groups with less differentially expressed proteins to begin with can have excessive false positives or false negatives if the transformation factor differed more than 0.3 units off from its true value.

To ensure the conservation of initial findings and using the TP, FP, and FN statistics accumulated from this analysis, we decided that the CI limit of the transformation factors for each protein should not exceed ± 0.3. At the 0.3 interval in both the positive and negative direction in both the MISC versus healthy and minimal COVID versus healthy analyses, at least 60% of the initial findings were conserved. Out of the 857 total modelable proteins by linear models, 686 had a slope CI of less than or equal to ± 0.3 (Fig. 3*C* and supplemental Table S4).

### Validation of Transformation Factors With Two Independent Cohorts

After creating a list of modellable proteins from the initial cohort, we tested the validity of the defined transformation factors and CI limits using two independent sets of matched serum/plasma patient samples. The goal of the validation was to assess whether the transformation factors remained robust across different patient phenotypes and sample processing conditions.

The first validation cohort included 40 matched serum and plasma samples from pediatric patients who had received CAR-T cells for the treatment of B-ALL. The Olink Target 96 Immune-Oncology panel, which measures 92 proteins using the same assay and technology as the larger Olink Explore panel, was utilized. The smaller panel was run locally to confirm the absence of machine, kit, or human biases that could affect the protein-specific transformation factors.

**Fig. 4:**
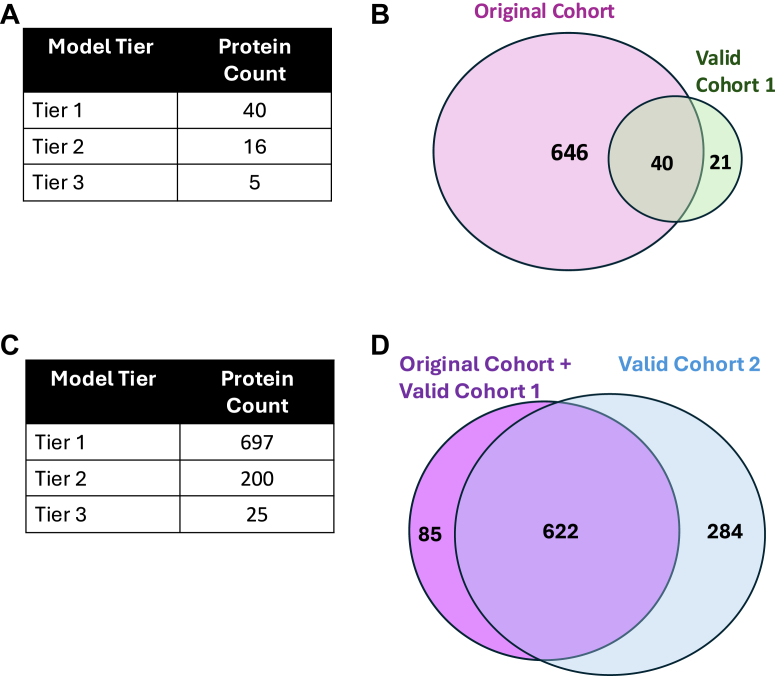
**Models of proteins in validation datasets**. *A*, breakdown of proteins from the first validation dataset based on linear modeling tiers. *B*, overlap of modelable proteins between the original cohort and the first validation cohort. *C*, breakdown of proteins from second validation dataset based on linear modeling tiers. *D*, overlap of modelable proteins from the original, first validation, and second validation cohorts.

A Pearson correlation test showed that 64 proteins (70%) had a correlation value of 0.5 or greater between their serum and plasma values, reaffirming the validity of using linear models for these proteins. After fitting all 92 proteins into the three defined tiers of linear models, 61 proteins (66%) were categorized as modelable, as shown in Figure 4*A* and supplemental Table S5). Similar patterns of variance and overall expression were observed in this cohort as in the initial cohort. All modelable proteins measured had a slope CI between 0 and 0.26. Out of the 92 proteins in the panel, 51 were categorized as modelable based on the initial cohort. When refitting these 51 proteins using data from the second cohort, 40 proteins (78%) had an R-squared value of 0.5 or above (Fig. 4*B*). Of the 11 proteins not successfully modeled in the second cohort, 10 had lower variance in either their serum or plasma concentrations, suggesting that the homogeneity of this cohort may have affected our ability to detect the linear relationship. Comparing the slopes of the modelable proteins from both cohorts, 35 (88%) had slope values differing by less than or equal to 0.3, validating the reproducibility of our transformation factors.

The second validation cohort consisted of Olink Explore data from 40 matched serum and plasma samples collected from adult patients on extreme ends of the BMI spectrum, previously reported by Wik *et al*. (19). Plasma samples in this cohort were collected in EDTA plasma tubes, unlike the original and first validation cohorts. Additionally, this cohort consists of all adult patients. This validation tested the robustness of the transformation factors regardless of these biological and sample processing differences.

Assessing the linearity of serum-plasma protein measurements using Pearson correlation, 864 (59%) proteins had a correlation coefficient of ≥0.5, closely aligning with correlation patterns observed in our original and first validation cohorts. Applying our tiered modeling approach to this validation cohort resulted in 697 proteins modeled in Tier 1, 200 proteins in Tier 2, and 25 proteins in Tier 3, totaling 922 modelable proteins (63%) as shown in Figure 4*C* and supplemental Table S6). Of these, 906 had confidence intervals (CIs) within ±0.3, and 622 proteins overlapped with the 707 proteins previously identified as modelable in our original and first validation cohorts (Fig. 4*D*). The 85 proteins classified as modelable in the prior cohorts but not in this new validation cohort exhibited lower variance in this cohort, potentially explaining their lack of modelability here. Importantly, when directly comparing transformation factors between cohorts, 551 proteins demonstrated consistency within the acceptable ±0.3 CI threshold.

### Final List of Transformation Factors

A total of 686 proteins were initially identified as modelable based on our original cohort. To independently validate these transformation factors, we tested a subset of 51 using a second cohort. The validation showed that 40 out of the 51 proteins (78%) maintained their modelability. Additionally, this second cohort identified 21 proteins as modelable that had not been classified in the original analysis, expanding the total number of modelable proteins to 707. To further validate our methodology in a non-pediatric context utilizing the full set of proteins measured by the Olink Explore panel, we applied our modeling approach to data generated by Wik *et al*. (19). Comparing R-squared values, confidence intervals (CIs), and transformation factor magnitudes, we found that 551 of the 707 proteins (78%) were consistently validated across all three cohorts, underscoring their broad applicability. For proteins modeled in multiple cohorts, we selected the transformation factor derived from the linear model exhibiting the highest R-squared value, thus ensuring optimal accuracy and reliability. This final list of 551 transformation factors is reported in supplemental Table S7.

## Discussion

Plasma and serum are common mediums to study various biological disease markers. Both mediums are used in a variety of technologies such as proteomics, transcriptomic, and metabolomics to compare biological profiles across disease groups (6, 7, 9, 15, 30). Although previous studies have tried to directly compare protein concentrations across mediums, the applicability of such comparisons typically depends on technology, the biology of the proteins themselves, and pre-analytical conditions related to sample collection and processing.

In this study, we demonstrated that a substantial number of proteins measured using Olink technology exhibit strong linear relationships between serum and plasma. Leveraging these relationships, we used linear models to generate transformation factors to normalize serum to plasma protein concentrations. We then validated these factors in two independent cohorts, each differing notably in patient demographics, pediatric versus adult, and technical processing variables. Specifically, the first validation cohort employed a smaller, targeted Olink panel, while the second utilized different plasma collection tubes, EDTA versus lithium heparin which was used in our original cohort.

Despite these demographic and technical variations, we successfully validated the transformation factors for 551 proteins across all three cohorts, demonstrating their robustness and reproducibility within established standard error limits. These findings highlight that certain proteins exhibit stability across demographic and technical variations, reinforcing the general applicability of our developed transformation factors. Our analyses also revealed that greater variability within serum and plasma sample subsets, and thus a more biologically heterogeneous patient cohort, is a contributing factor for successfully developing protein models. We have detailed the various tiering, strengths of correlation, and reproducibility across cohorts we used so that future users of our transformation factors may choose more or less than the 551 we present as our final set based on their needs for stringency. The full data needed to make these choices may be found in the various supplemental data tables.

Together, these findings suggest that while a substantial number of proteins demonstrate robust cross-cohort modeling, others exhibit cohort-specific patterns of transformability. This specificity likely arises from the variance of protein quantification observed within each dataset and/or the underlying biological characteristics and sample collection of each patient cohort. Thus, while some proteins may show robust, cohort-independent transformability, as demonstrated by the 551 proteins validated across all cohorts, other proteins, including the ones only modelable in our validation cohorts, may only be reliably modeled within particular clinical or biological contexts.

Importantly, these findings underscore the practical utility of our approach for real-world proteomic studies using banked samples. By normalizing data obtained from varied sample types, researchers can effectively expand the size of their cohorts, thus enhancing study power and applicability. This approach is especially advantageous in pediatric research, where obtaining blood samples can be particularly challenging and limited. Researchers intending to apply our method to integrate previously generated datasets must consider batch effects inherent to technology. In such cases, our methodology is applicable when bridging samples, i.e., identical samples measured across datasets, are available to adequately account for and normalize these batch differences after transforming the serum to plasma protein quantifications.

To fully realize the translational potential of these transformation factors, future work should prioritize validating our transformation factors across additional disease populations and clinical contexts to further confirm their broad applicability and robustness. As the scope and capacity of Olink assays continue to expand, applying our methodology to newly developed larger Olink panels could uncover more proteins exhibiting consistent linear relationships between serum and plasma. Furthermore, expanding this approach to other proteomic platforms could substantially enhance data integration efforts. In conclusion, this study successfully identified robust transformation factors for 551 proteins measured using Olink. By providing a method to integrate data across different studies, our work supports the development of more comprehensive biomarkers and the understanding of disease mechanisms across diverse patient cohorts, enhancing collaborative and translation research efforts.

## Data Availability

Analyses for our second validation cohort was completed by Dr Anders Mälarstig and his team. For access to that data please request via a data processing agreement with Karolinska Institute (Anders Mälarstig, anders.malarstig@ki.se, Karolinska Institute). Data generated within the Children’s Hospital of Philadelphia can be found in Supplemental tables S1-S2 and code used to generate the models and results presented in the manuscript can be found on GitHub (https://github.com/shraimrawan/Olink.git).

## Supplemental data

This article contains supplemental data.

## Conflicts of interest

The authors declare the following financial interests/personal relationships which may be considered as potential competing interests: David T. Teachey receives research funding from BEAM Therapeutics and NeoImmune Tech. David T. Teachey serves on advisory boards for Janssen, Sobi, Jazz, and Servier. David T. Teachey has patents (US11747346) and patents pending on CAR-T. Edward M. Behrens receives research funding from AB2Bio. Edward M. Behrens serves as a consultant for Sobi and Pharming. Anders Mälarstig and Erin Macdonald-Dunlop are employees of Pfizer Research and Development.
